# Novel mutations in the 3-box motif of the BACK domain of KLHL7 associated with nonsyndromic autosomal dominant retinitis pigmentosa

**DOI:** 10.1186/s13023-019-1275-2

**Published:** 2019-12-19

**Authors:** Jin Kyun Oh, Jose Ronaldo Lima de Carvalho, Young Joo Sun, Sara Ragi, Jing Yang, Sarah R. Levi, Joseph Ryu, Alexander G. Bassuk, Vinit B. Mahajan, Stephen H. Tsang

**Affiliations:** 1 Department of Ophthalmology, Columbia University Medical Center, New York, NY USA; 2 State University of New York at Downstate Medical Center, Brooklyn, NY USA; 3 Department of Ophthalmology, Empresa Brasileira de Servicos Hospitalares (EBSERH) - Hospital das Clinicas de Pernambuco (HCPE), Federal University of Pernambuco (UFPE), Recife, Brazil; 4 Department of Ophthalmology, Federal University of São Paulo (UNIFESP), São Paulo, Brazil; 5 Omics Laboratory, Byers Eye Institute, Stanford University, Palo Alto, CA USA; 6 Department of Pediatrics, University of Iowa, Iowa City, USA; 7 Department of Neurology, University of Iowa, Iowa City, USA; 8 Veterans Affairs Palo Alto Health Care System, Palo Alto, CA USA; 9 Department of Pathology & Cell Biology, and Columbia Stem Cell Initiative, Columbia University Medical Center, 635 West 165th Street, Box 212, New York, NY 10032 USA

**Keywords:** Kelch-like protein 7, Inherited retinal dystrophy, Retinitis pigmentosa, Autosomal dominant, Kelch

## Abstract

**Background:**

Mutations in the Kelch-like protein 7 (*KLHL7)* represent a recently described and, to date, poorly characterized etiology of inherited retinal dystrophy. Dominant mutations in *KLHL7* are a cause of isolated, non-syndromic retinitis pigmentosa (RP). In contrast, recessive loss-of-function mutations are known to cause Crisponi or Bohring-Opitz like cold induced sweating syndrome-3 (BOS-3). In this study, the phenotype and progression of five unrelated patients with *KLHL7* mediated autosomal dominant RP (adRP) are characterized. Clinical evaluation of these patients involved a complete ophthalmic exam, full-field electroretinography (ffERG), and imaging, including fundus photography, spectral domain optical coherence tomography (SD-OCT), short wavelength fundus autofluorescence (SW-AF), and near-infrared fundus autofluorescence (NIR-AF). Molecular diagnoses were performed using whole-exome sequencing or gene panel testing. Disease progression was monitored in three patients with available data for a mean follow up time of 4.5 ± 2.9 years. Protein modeling was performed for all variants found in this study in addition to those documented in the literature for recessive loss-of-function alleles causing Crisponi or Bohring-Opitz like cold-induced sweating syndrome.

**Results:**

Genetic testing in three patients identified two novel variants within the 3-box motif of the BACK domain: c.472 T > C:p.(Cys158Arg) and c.433A > T:p.(Asn145Tyr). Clinical imaging demonstrated hyperautofluorescent ring formation on both SW-AF and NIR-AF in three patients, with diffuse peripheral and peripapillary atrophy seen in all but one case. SD-OCT demonstrated a phenotypic spectrum, from parafoveal atrophy of the outer retina with foveal sparing to widespread retinal thinning and loss of photoreceptors. Incidence of cystoid macular edema was high with four of five patients affected. Protein modeling of dominant alleles versus recessive loss-of-function alleles showed dominant alleles localized to the BTB and BACK domains while recessive alleles were found in the Kelch domain.

**Conclusions:**

We report the phenotype in five patients with *KLHL7* mediated adRP, two novel coding variants, and imaging biomarkers using SW-AF and NIR-AF. These findings may influence future gene-based therapies for adRP and pave the way for mechanistic studies that elucidate the pathogenesis of *KLHL7*-mediated RP.

## Background

Retinitis pigmentosa (RP) is characterized by the degeneration of rod photoreceptors followed by cone photoreceptors [1–4]. The disorder typically presents with symptoms of poor night vision and progressive tunneling of the visual field. Estimated to affect approximately 1 in 4000 individuals, RP is one of the most common and irreversible causes of blindness worldwide [1–4]. To date, over 80 genes have been implicated in the development of this condition, and new genes are continually being added to this list [5]. Currently, the most promising method of treatment for RP is gene-specific; thus, the natural history and identification of outcome measurements in clinical trials must be characterized for each genetic etiology of the disorder. While some genes commonly implicated in RP are well understood, those affecting smaller populations of RP patients present a more difficult challenge.

Heterozygous mutations in the Kelch-like protein 7 (*KLHL7,* OMIM# 611119) were first associated with autosomal dominant retinitis pigmentosa (adRP) in 2009 by Friedman et al. and fall under the lesser understood category of RP genes [6]. Investigations of KLHL7 function have since demonstrated that KLHL7 works primarily as an E2-ubiquitin intermediate receiver for the Cullin-based E3 ligase, Cul3, in the ubiquitin-proteasome degradation pathway (UPP) [6–9]. In the retina, failure of UPP function may lead to the accumulation of toxic substrates in the photoreceptor cells [10].

The structure of KLHL7 has been well characterized and consists of three functional domains: BTB (Bric-a-brac, Tramtrack, and Broad Complex), BACK, and Kelch [6–9]. The BACK domain bridges the BTB domain and Kelch domain and has a structural motif called a 3-box motif at its N-terminus. The 3-box motif forms a 16 Å deep cleft in combination with the C-terminus of the BTB domain that plays a key role in the Cullin-KLHL E3 ligase complex formation by recognizing the N-terminal tail of Cullin [9]. The Kelch domain has six Kelch repeats, forming the “blades” of a β-propeller structure. Each blade is composed of an anti-parallel β-sheet formed by four β-strands. The intra-blade loops connecting the second and third β-strand of each blade and the loops bridging the neighboring blades (inter-blade loops) determine the substrate specificity of the Kelch domain [9]. One in vitro study suggests that BTB and BACK deletions abolish KLHL7 and Cul3 interactions, indicating that both BTB and BACK are integral for Cul3 protein binding [7]. Prior reports of *KLHL7*-mediated adRP have all involved mutations of the BACK domain [6, 11, 12]. In contrast, recessive loss-of-function alleles in *KLHL7*, which cause Crisponi and Bohring-Opitz cold-induced sweating syndrome-3 (BOS3), occur in the Kelch or BTB domain [13–16]. This domain-dependent effect on phenotype has been described in a number of other retinal dystrophies, including those associated with *RP1* and *RHO*, which exhibit similar domain-dependent effects on protein function, disease presentation, and patterns of inheritance [17–19].

The molecular function of KLHL7 is well described, but the phenotypic description of adRP associated with this gene is limited. We present five adRP patients with confirmed *KLHL7* mutations, three of whom carried novel mutations.

## Results

### Clinical summary

**Fig. 1:**
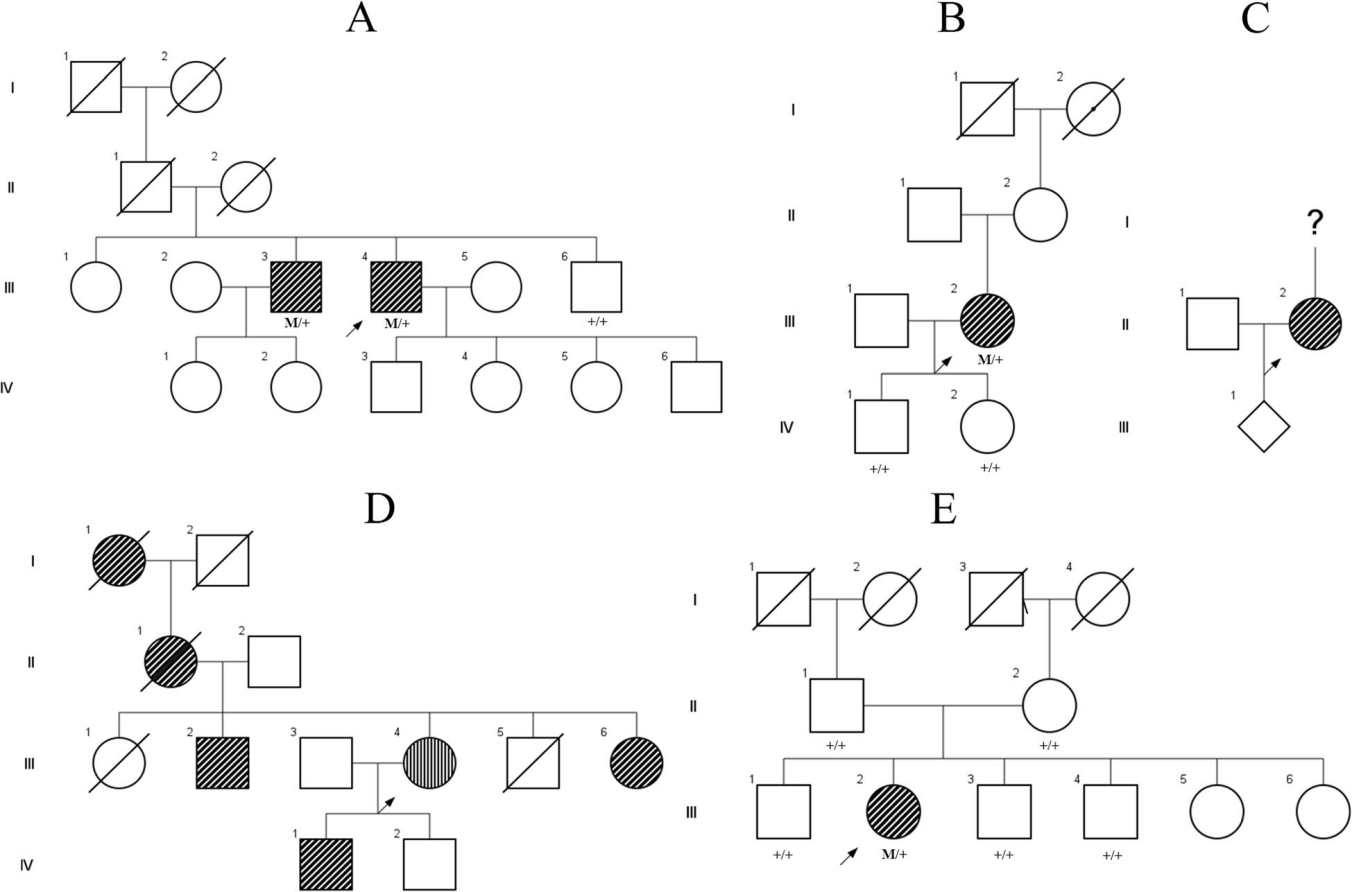
Pedigrees of Patients with Mutation in the 3-box motif of the BACK domain of KLHL7. Pedigrees of all five patients with mutations in the 3-box motif of the BACK domain in KLHL7 were obtained and segregation analysis of the variants within the pedigrees was performed as obtainable. **a** Segregation of the variant in the family pedigree of P1 was seen in the proband and one affected brother and was not seen in one unaffected brother. **b** The variant identified in P2 was not identified on testing of the two unaffected children. **c** Segregation analysis was not possible in P3. **d** Family history in P4 was consistent with a dominant pattern of inheritance. **e** Segregation of the variant in the family pedigree of P5 suggested that the mutation developed de novo given the absence of the variant in both parents and three unaffected siblings

Genetic testing identified five unrelated RP patients with variants in the *KLHL7* gene who underwent clinical evaluation. A summary of the demographic, clinical, and genetic information of these patients is found in Table 1. The family pedigrees for each patient are shown in Fig. 1. Two patients presented with progressive nyctalopia (P1, P4). Three patients (P1, P3, P5) were examined longitudinally with a mean follow up time of 4.5 ± 2.9 years. Six eyes from 4 patients (P1, P3, P4, P5) had cystoid macular edema (CME) on fundus examination on initial presentation.

**Table 1:** Patient demographics for 5 *KLHL7*-mediated Retinitis Pigmentosa Patients

ID - Gender	Age (Age at diagnosis)	Ethnicity	Variant	Retinal Pigment Migration	CME	BCVA at first visit	BCVA at most recent visit
P1 - M	68 (45)	Scottish	c.472 T > C: p.(Cys158Arg)* heterozygous	Yes, up to arcades	OS	20/25, 20/30	20/63, 20/32
P2 - F	49 (21)	Irish	c.458C > T: p.(Ala153Val) heterozygous	Yes, up to arcades	None	20/150, 20/150	20/CF @ 2 ft., 20/CF @ 4 ft
P3 - F	39 (32)	Korean	c.433A > G: p.(Asn145Asp) heterogyzous	Yes, up to arcades	OU	20/20, 20/25	20/30, 20/30
P4 - F	59 (32)	Caucasian (unspecified)	c.472 T > C: p.(Cys158Arg)* heterozygous [SNRNP200 c.983-8G > A heterozygous]	Yes, up to arcades	OS	20/50, 20/40	
P5 - F	39 (33)	Irish	c.433A > T: p.(Asn145Tyr)* heterozygous	Yes, up to arcades	OU	20/30, 20/30	20/40, 20/40

*CME* Cystoid macular edema, *BCVA* Best corrected visual acuity, *OD* Right eye, *OS* Left eye, *OU* Both eyes,

*: Novel

### Imaging & Progression

**Fig. 2:**
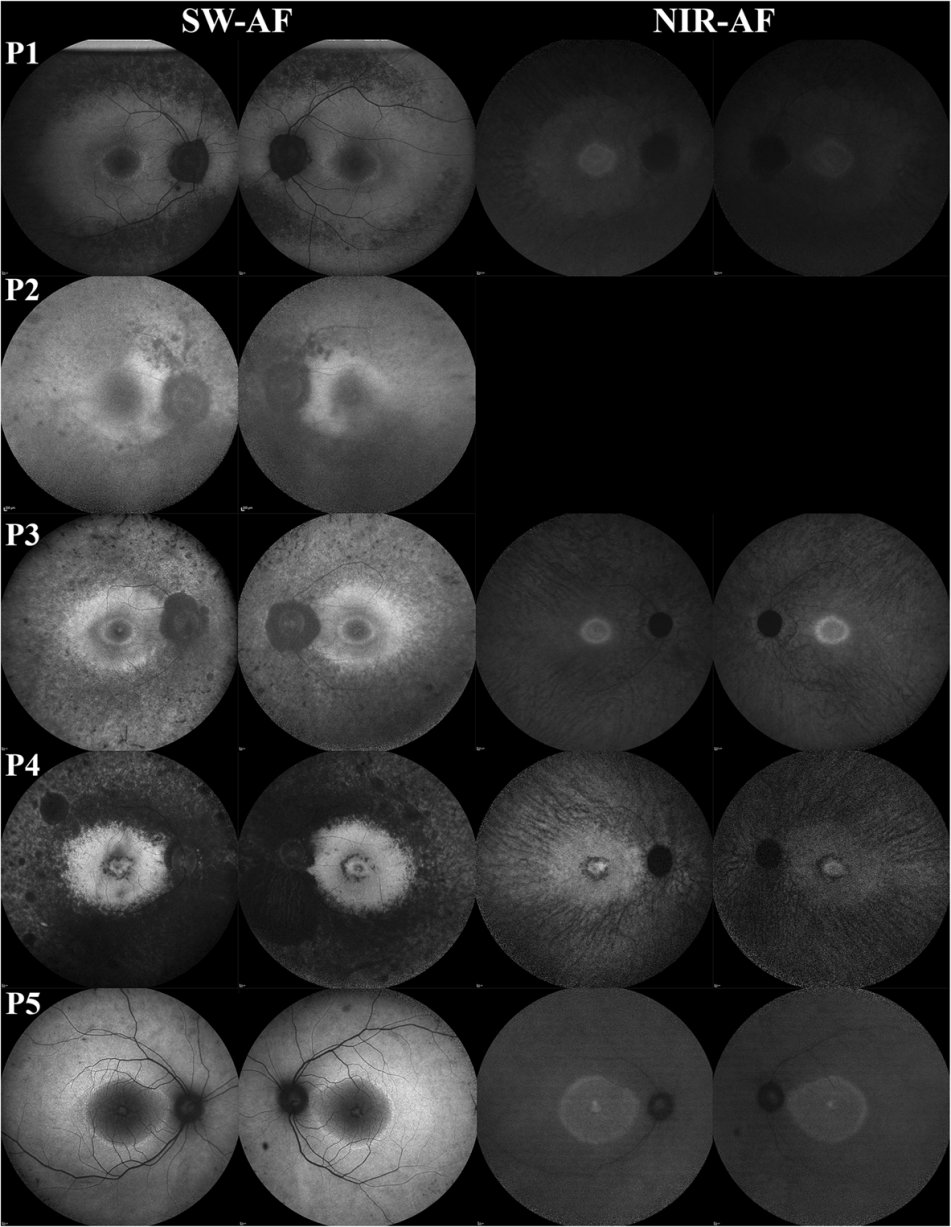
Short Wavelength and Near Infrared Autofluorescence Findings of *KLHL7* mutation. Short-wave (SW-) and near-infrared autofluorescence (NIR-AF) imaging demonstrated a hyperautofluorescent ring in P1, P3, and P5 with diffuse peripheral hypoautofluorescence and peripapillary atrophy. P2 and P4 presented with more severe phenotypes with absent hyperautofluorescent rings and dense peripheral and peripapillary atrophy. P4 in particular was noted to have an additional smaller ring of atrophy surrounding the fovea

On SW-AF and NIR-AF, patients P1, P3, and P5 showed central hyperautofluorescent rings and diffuse peripheral hypoautofluorescence with peripapillary atrophy (Fig. 2). P2 was found to have similar peripheral findings and peripapillary atrophy on SW-AF but without a hyperautofluorescent ring. P4 showed a small central island of spared retina surrounded by a ring of atrophy and dense peripheral and peripapillary atrophy on SW-AF and NIR-AF.

**Fig. 3:**
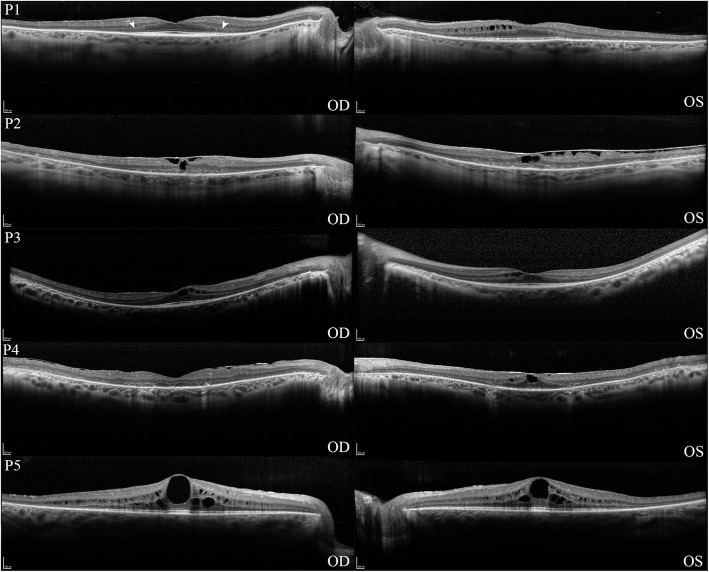
Central EZ preservation in Optical Coherence Tomography of Patients with *KLHL7* mutation. Spectral domain optical coherence tomography of both eyes in five patients with *KLHL7* mutation demonstrated a pattern of parafoveal atrophy of the outer retinal layers (white arrows) in seven out of ten eyes (P1, P2, P4, P5). Both eyes of P2 and the right eye of P4 demonstrated global thinning of the inner and outer nuclear layers with loss of photoreceptors. Six of ten eyes (P1, P3, P4, P5) were noted to have differing degrees of cystoid macular edema. P2 was found to have bilateral foveal traction secondary to epiretinal membrane formation

Spectral domain optical coherence tomography (SD-OCT) was performed. Patients P1, P3, and P5 had centrally well-preserved retinal architecture with parafoveal atrophy of the outer retina, including the outer nuclear layer (ONL), external limiting membrane (ELM), ellipsoid zone length (EZ), and cone outer segment tips line (COST, Fig. 3). CME was observed in the inner and outer nuclear layers of these three patients. P2 was found to have both parafoveal and foveal thinning of the outer retinal layers with complete loss of the ELM, EZ, and COST lines. Bilateral macular traction secondary to epiretinal membrane formation was also noted. P4 had asymmetric disease on SD-OCT with a foveal sparing in the left eye similar to P1, P3, and P5. There was diffuse thinning and loss of the outer retina in the right eye similar to P2.

Disease progression was evaluated in the three patients (P1, P3, P5) who had quantifiable EZ line and hyperautofluorescent rings. Intraclass correlation coefficient (ICC) was 0.99 (*p* < 0.001) for all parameters, suggesting high test-retest reliability between the two graders. Constriction of the EZ line and hyperautofluorescent rings were seen in all three patients. The mean rates of progression per year were -101 μm (− 2.8%) for EZ line, − 86 μm (− 2.0%) for horizontal diameter, and -82 μm (− 2.3%) for vertical diameter.

### Electroretinography

**Fig. 4:**
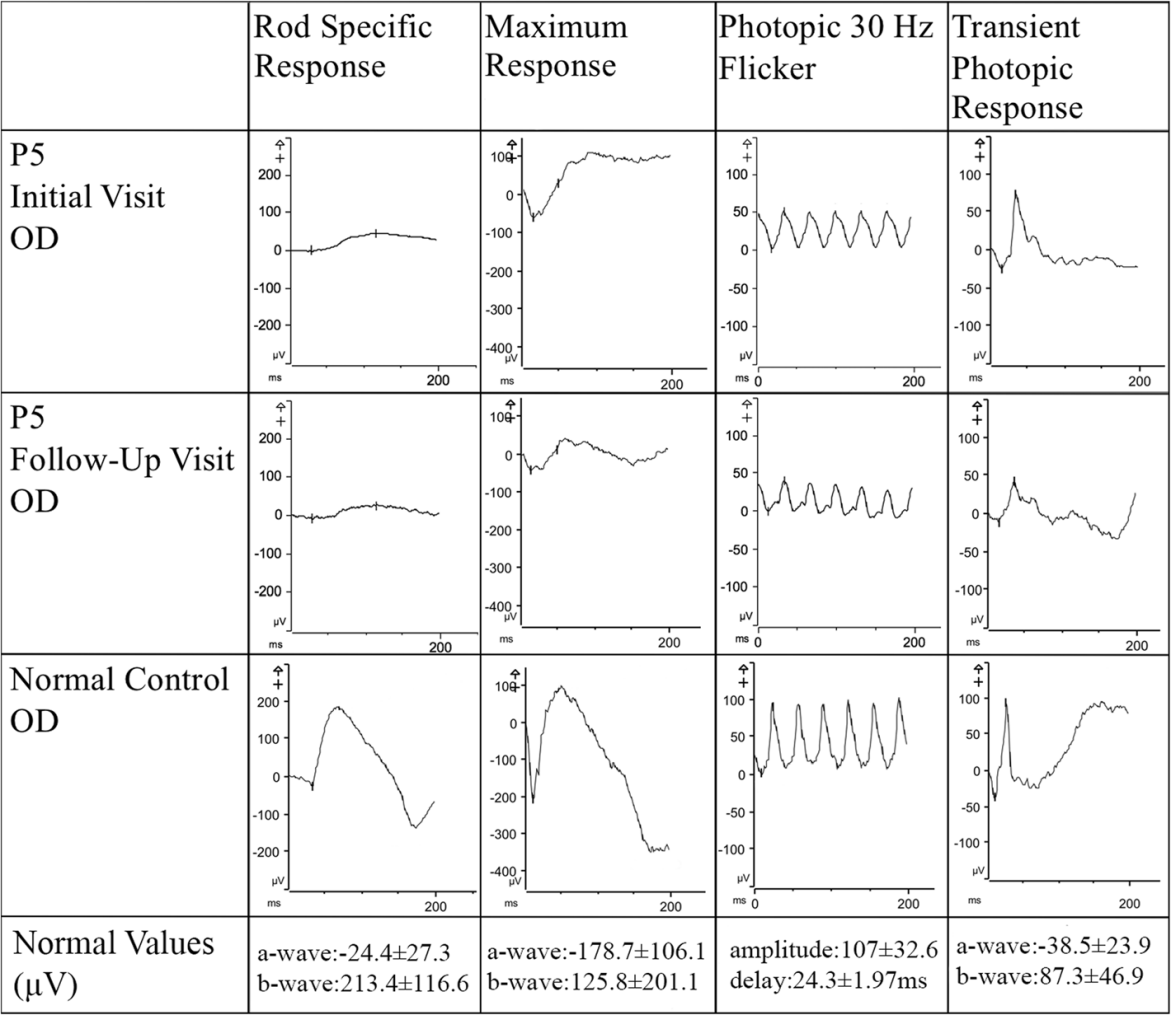
Rod Cone Dysfunction in Full Field Electroretinogram Findings of Patient 5. Full-field electroretinogram findings of the right eye of P5 across two visits separated by 2 years demonstrated a slow decrease in both scotopic rod-specific and photopic 30 Hz flicker suggestive of slow disease progression. Normal values were demonstrated through an age-matched control patient

All patients underwent full-field electroretinogram (ffERG) testing and were found to have a rod-cone pattern of degeneration (Table 2). Scotopic rod-specific b-waves were extinguished in four of the five patients (P1, P2, P3, P4) while P5 maintained some rod function in both eyes (Fig. 4). Three of the four patients with extinct rod-function (P2, P3, P4) had diminished cone function on 30 Hz flicker. Patients P1 and P5 maintained relatively spared cone function as observed on their 30 Hz flicker test.

**Table 2:** Full-Field Electroretinography Evaluation of *KLHL*-related Retinitis Pigmentosa Patients

ID	30 Hz Flicker OD (μV)	30 Hz Flicker Implicit Times OD (ms)	30 Hz Flicker OS (μV)	30 Hz Flicker Implicit Times OS (ms)	Scotopic Rod-Specific B-wave OD (μV)	Scotopic Rod-Specific B-wave OS (μV)
P1	30	37	42	35	Extinguished	Extinguished
P2	2	40	1.4	40	Extinguished	Extinguished
P3	12^BA^	40^BA^	8^BA^	40^BA^	Extinguished^BA^	Extinguished^BA^
P4	2	33	3.5	36	Extinguished	Extinguished
P5	52	35	37	35	48	21

*BA* Burian-Allen, *OD* Right eye, *OS* Left eye, *OU* Both eyes

### Variant identification

Two unrelated patients, P1 and P4, were found to be heterozygous for a novel missense variant c.472 T > C:p.(Cys158Arg) that was predicted to be damaging (Provean score: [− 10.93,-10.73], SIFT score: 0, PolyPhen: probably damaging, Mutation Taster: disease causing). Segregation analysis of the variant within the family pedigree of P1 confirmed the presence of the variant in both the patient and his affected brother and its absence in the unaffected brother (Fig. 1a). On panel testing, interestingly, P4 was also found to be heterozygous for c.983-8G > A, a splice variant in SNRNP200 (PolyPhen, SIFT, Mutation Taster not available). This variant was classified as one of uncertain significance as it has not been reported in public mutation databases such as gnomAD, HGMD, or Clinvar and there was insufficient evidence to suggest or rule out pathogenicity. Segregation analysis of the variant was recommended for this patient, but she was lost to follow up. P2 was identified as heterozygous for a previously reported pathogenic variant c.458C > T:p.(Ala153Val). Segregation analysis of the variant within the family pedigree of P2 demonstrated the pathogenic variant was absent in both unaffected children (Fig. 1b). The c.433A > G:p.(Asn145Asp) missense variant found in P3 has been reported once in a patient with a diagnosis of RP and is not found in common variant databases including ExAC, gnomAD, and the NHLBI Exome Variant Server Database [20]. It was predicted to be deleterious (score:-4.47) and damaging (score:0) by Provean and SIFT. As the patient was adopted, segregation analysis was not possible. A novel missense variant c.433A > T:p.(Asn145Tyr) was found in P5 and predicted to be damaging by PolyPhen-2 (score:1.0). Segregation analysis of the variant within the family pedigree of P5 demonstrated the absence of the variant in both parents as well as three unaffected siblings. The remaining two siblings were seen and evaluated by an outside retinal specialist and were endorsed to be unaffected. These findings suggest this variant is likely a de novo mutation, although the possibility of germline mosaicism cannot be ruled out. The absence of the disease or the variant in the siblings, however, suggest mosaicism is less likely.

### Protein modeling

**Fig. 5:**
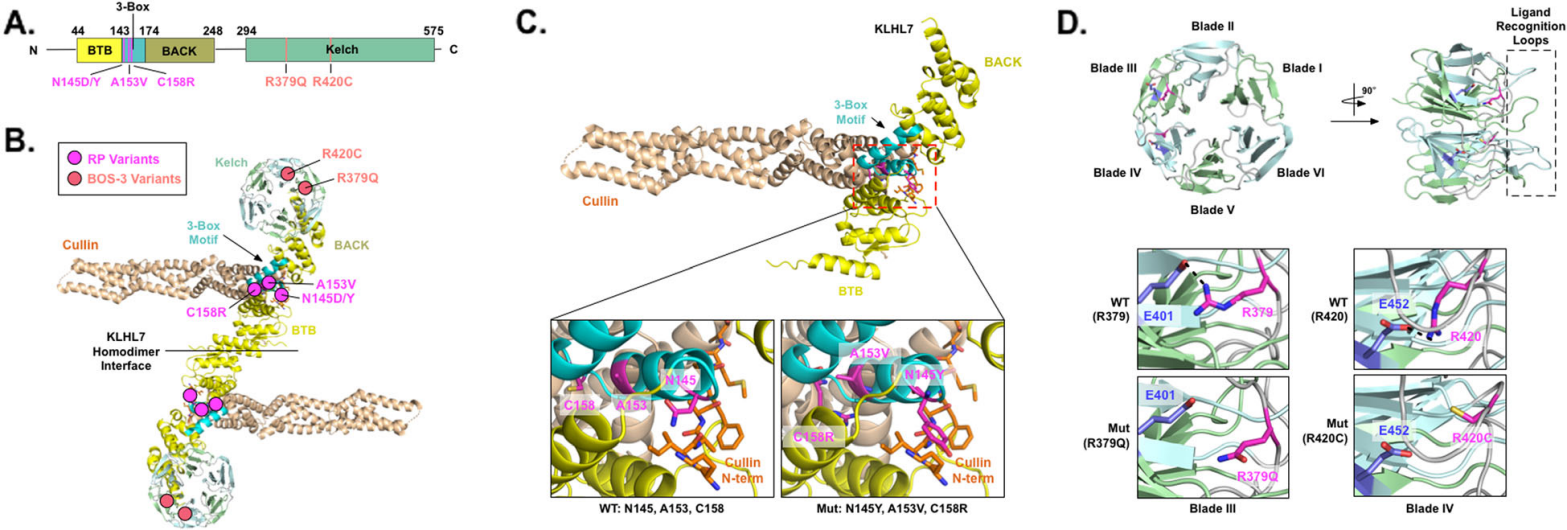
Structural modeling of KLHL7 demonstrates the domain-dependent correlation between genotype and phenotype. **a** A model of the domain topology of KLHL7 and coding variants are illustrated. The BTB domain is shown in the yellow box, the 3-box motif is shown in the blue box, the BACK domain is shown in the dark mustard box, and the Kelch domain is shown in the green box. Each RP variant is shown in magenta, and each BOS-3 variant is shown in pink. **b** Three-dimensional structure of the KLHL7 homodimer in complex with Cullin is shown based on the previously proposed model. Cullin is shown in orange, and each domain of KLHL7 is color-coded corresponding to panel A. The locations of each coding variant are shown in the circles. **c** Structural modeling of the KLHL7 BTB-BACK domain is shown in complex with Cullin. The bottom left panel shows a magnified view of wild-type (WT) KLHL7 3-box motif (cyan) interacting with the N-terminus of Cullin (orange). The bottom right panel shows a magnified view of mutations at the 3-box motif of KLHL7. Each mutation is shown in magenta. c.433A > T:p.(Asn145Tyr) disrupts the direct interaction of the motif with the N-terminus of Cullin, while c.458C > T:p.(Ala153Val) and c.472 T > C:p.(Cys158Arg) disrupt the unique structural conformation of the 3-box motif. **d** Structural modeling of mutations found at the Kelch domain of KLHL7 is illustrated. The Kelch domain is formed by six-β-blades forming a β-propeller structure. Each blade is numbered from the N-terminus. The dotted box shows the ligand recognition loops of the Kelch domain. Mutations are shown in magenta and the glutamates that make charge-charge interaction with the mutated arginines are shown in blue. These charge-charge interactions are lost upon mutation

A protein model of KLHL7 with its three associated domain features was generated (Fig. 5). The four *KLHL7* mutations identified in our cohort of adRP patients all localized to the BACK domain and more specifically to within the 3-box motif (Fig. 5b magenta circles & Fig. 5c lower panels). The 3-box motif is known to play a key role in Cullin-KLHL E3 ligase complex formation by recognizing the N-terminal tail of Cullin [9]. Modeling variants c.433A > T:p.(Asn145Tyr) and c.433A > G:p.(Asn145Asp) showed that they disrupted the direct interaction between the 3-box motif and the N-terminal tail of Cullin. Variants c.458C > T:p.(Ala153Val) and c.472 T > C:p.(Cys158Arg) disrupted the structural conformation of the motif itself. These effects on critical functional motifs may lead to poor recognition of the N-terminal tail of Cullin and diminish formation of the Cullin-KLHL E3 ligase complex. Thus, we surmise that the nonsyndromic RP phenotype is most likely due to defects in KLHL7-Cullin E3 ligase complex formation.

In contrast, modeling *KHLH7* mutations that are implicated in Crisponi and BOS3, c.1115G > A:p.(Arg372Gln) and c.1258C > T:p.(Arg420Cys), revealed that they were localized to the Kelch domain (Figure 5b pink circles) [12–15]. More specifically, these arginine amino acids were located at structurally analogous positions in blades III and IV within the inter-blade loop. Functionally, they make charge-charge interactions with their corresponding glutamates (Glu401 and Glu452) at the third β-strand of each blade (Figure 5d WT panels). The arginine-glutamic acid charge-charge interaction normally latches the neighboring blades together, which ultimately contributes to the proper tertiary structure (β-propeller) folding of the Kelch domain. However, in the BOS3 variant models, these interactions are disrupted by the disease-associated amino acid substitutions (Figure 5d Mut panels). Thus, we believe that the BOS3 patient phenotype is likely due to the reduced stability of the Kelch domain.

## Discussion

Genetic etiologies of retinal dystrophy causing both autosomal dominant and recessive disease have been described in the literature and include *RHO*, *RP1, BEST1*, *GUCY2D, RAX2,* and *RPE65* [17–19, 21–24, 25]. Domain-dependent differences in dominant and recessive disease have been suggested for *RP1,* but the structural correlation of variants with disease phenotype in the other genes is still a topic of investigation [17–19, 21]. Prior studies of *KLHL7-*mediated RP suggest differing onset of retinal degeneration in autosomal dominant and recessive disease. Age of onset in patients with the recessive disorder was found to occur prior to 6 years of age, while the mean age of onset of isolated retinal disorder for those with a dominant mutation was found to be 53, which is later than the average for adRP [12–16]. In our study of 5 unrelated heterozygous patients, we found a mean and median age of symptomatic onset at 32.6 years and 32 years respectively, which was earlier than both the average for adRP and prior reports in the literature [6, 11, 12, 26]. Based on longitudinal data and subjective reports from four of our patients, disease progress appeared slow in three of the patients, and visual acuity remained stable. One patient (P2) was diagnosed at a young age and demonstrated a late and rapid progression atypical of the identified variant as discussed below.

The phenotypic spectrum seen on SD-OCT imaging of the patients varied between a milder parafoveal atrophy of the outer retina with foveal sparing to pan-retinal atrophy of the outer retina with widespread loss of photoreceptors. This is consistent with prior description of SD-OCT findings in *KLHL7*-mediated adRP [12]. Visual acuity correlated well with phenotypic severity, with vision ranging from between 20/20 to 20/40 for the milder phenotype to 20/CF in more advanced disease. The EZ line seen in early disease progressively shortened in width over time, suggesting that it may be of potential use as an outcome measurement only in early disease stage. The rate of loss was identified to be 101 μm per year, which was significantly less than the rate of loss caused by other genes associated with adRP and X-linked RP, suggesting that disease progression may be slower in *KLHL7* adRP than in other forms of adRP [27, 28]. CME was found at a higher rate in our cohort in comparison to reports by Hugosson et al. (1/11 patients) and Wen et al. (1/5 patients) and was notable in six of seven eyes that had foveal sparing on SD-OCT, suggesting that CME may be a more common finding in early stages of disease [11, 12].

Both SW-AF and NIR-AF imaging of our patients demonstrated the presence of a hyperautofluorescent ring in the more phenotypically mild patients, P1, P3, and P5. This hyperautofluorescent ring was similar to those typically seen in patients with RP and correlated structurally with the boundary of intact inner and outer segment junction [29]. Progressive decrease in the vertical and horizontal diameter of the hyperautofluorescent ring seen in P1, P3, and P5 suggested that in early stage disease, these measurements may have potential use as outcome measurements in the future treatment of this condition. The rate of loss of vertical and horizontal diameter of the ring in *KLHL7* adRP was similarly slower than that reported in other causes of adRP; however, evaluation of a larger cohort of patients will help corroborate these findings [27, 28]. In more structurally advanced disease as seen in P2 and P4, the hyperautofluorescent ring is lost and can no longer be used as a potential outcome measurement for treatment.

The functional loss seen on ffERG in this cohort suggests complete loss of rod photoreceptor function in the late forties, which is earlier than in previously described reports [12]. Similarly, the literature describes a loss of 3% per year in 30 Hz flicker amplitude on ffERG as compared to the average 10% per year typically attributed to RP [30]. In the case of P5, the 30 Hz flicker amplitude declined at annual rates of 11 and 6% in the right and left eye respectively. The earlier extinction of rod photoreceptor function and the accelerated decline in cone photoreceptor seen in our cohort suggest that *KLHL7* mediated adRP may cause functional loss at a speed more similar to other forms of RP than previously described [12, 30].

Despite the same novel missense variant, c.458C > T:p.(Ala153Val), being identified in P1 and P4, their age of onset and phenotypic presentation were varied. Earlier onset and a more severe phenotype were seen on all imaging modalities in P4 as compared to P1. This difference may be attributed to phenotypic variability associated with this novel variant. However, P4 was also found to have a novel variant of undetermined significance in *SNRNP200*, another gene implicated in adRP, suggesting the possibility of digenic contribution of the heterozygous mutations as a cause of the more severe phenotype. The novel missense variant, c.433A > T:p.(Asn145Tyr), identified in P5 occurred at the same locus as the previously reported pathogenic mutation c.433A > G:p.(Asn145Asp) seen in P3. P5 presented with a milder phenotype on imaging and ffERG as compared to P3, despite a nearly identical age of onset and age at evaluation. This suggested that the effects of these variants on protein function may differ in severity. An alternative explanation for the difference may be attributed to germline mosaicism, which is known to cause milder phenotypes [31]. Further studies will be required to better elucidate genotype-phenotype correlations for these variants.

P2 presented with the variant c.458C > T:p.(Ala153Val), which was one of the first identified pathogenic variants in *KLHL7* [6, 11, 12]. However, the patient presented with a severe phenotype on imaging and a visual acuity of 20/CF at the most recent visit which were atypical of other previously described cases in the literature [11, 12]. Hugosson et al. and Wen et al. described a total of seven patients with this variant, six of whom presented with visual acuities between 20/20 and 20/60 [11, 12]. One patient was found to have acuity of 20/200 but was also evaluated at 69 years of age. The disease severity seen in P2 suggested the possibility of phenotypic variability associated with this variant. Evaluation of a larger cohort of patients with this variant will help corroborate genotype-phenotype correlations.

Structural analysis of the variants seen in our cohort when compared to reported recessive loss-of-function alleles suggests a domain-dependent correlation between genotype and phenotype. Mutations in the 3-box motif of the BACK domain appear to cause interruptions to the assembly of Cul3 ligase, and, consequently, the UPP degradation pathway in a dominant negative fashion [ 6, 7, 10, 13, 15]. Interruptions of the UPP degradation pathway have been implicated in other genetic etiologies of RP, such as *TOPORS* mediated adRP and the p.Pro23His variant in *RHO* mediated adRP [32]. In contrast, recessive loss-of-function alleles in the Kelch domain lead to instability in tertiary structure and more severe and syndromic disease. This allelic location-dependent difference in disease is seen in other genes that cause both dominant and recessive disease, including those caused by *RHO* and *RP1*. As such, this topic is a source of interest as treatment of dominant negative mutations presents a different challenge than the gene supplementation typically used to treat recessive loss-of-function disease [33]. Further studies are needed to correlate the effects of protein change on the phenotype seen in this condition.

## Conclusions

Mutations in *KLHL7* are a recently described etiology of adRP that have, to date, been reported infrequently in the literature. In this study, we characterized for the first time the SW-AF and NIR-AF findings of five patients with documented *KLHL7* mutations and described three patients with novel variants that cause amino acid substitutions in the 3-box motif of the BACK domain of the protein. These findings may influence future gene-based therapies for adRP as well as pave the way for mechanistic studies that elucidate the pathogenesis of *KLHL7*-mediated RP.

## Methods

### Subjects

Retrospective analysis of five unrelated patients with documented mutations in *KLHL7* was performed. All patients were evaluated at the Edward S. Harkness Eye Institute at Columbia University Medical Center. Patient consent was obtained as per Columbia University Institutional Review Board-approved protocol AAAR8743 and all procedures were reviewed and in accordance with the tenets of the Declaration of Helsinki. The data presented in this study was procured through retrospective chart review and was not identifiable to any individual patient.

### Examination and imaging

Ophthalmic examination included measurement of best corrected visual acuity followed by dilation with topical tropicamide (1%) and phenylephrine hydrochloride (2.5%), fundus examination, fundus photography, SD-OCT, SW-AF (488 nm excitation, barrier filter transmitted light from 500 to 680 nm, 55° × 55° field autofluorescence), and NIR-AF (787 nm excitation, 830 nm emission, 55° × 55° field). SD-OCT, SW-AF, and NIR-AF were acquired using a Spectralis HRA + OCT (Heidelberg Engineering, Heidelberg, Germany). Ultra-widefield color fundus photography was performed using an Optos 200 Tx (Optos, PLC, Dunfermline, United Kingdom).

### Progression

Disease progression was determined between initial and most recent visits using outcome measurements of ellipsoid zone line length on SD-OCT as well as vertical diameter and horizontal diameter on SW-AF. Both vertical and horizontal diameter were measured as the longest distance between the outer borders of the superior-inferior and temporal-nasal ring respectively. Two independent graders (J.O. and J.R.L.) assessed these outcomes in both eyes using a built-in measurement tool on the Spectralis HRA + OCT software. Statistical analysis of ICC and descriptive statistics for disease progression were determined using R statistical software version 3.61 (Vienna, Austria).

### Electroretinography

Full-field ERG of scotopic and photopic states was performed in each eye of all five patients using DTL electrodes and Ganzfeld stimulation on a Diagnosys Espion Electrophysiology System (Diagnosys LLC, Littleton, MA, USA) according to international standards. BA contact lens electrodes were used when examination with DTL electrodes did not produce adequate waveforms.

### DNA analyses

DNA was isolated from peripheral blood of each patient for analysis. Two patient samples (P1 and P3) underwent whole exome sequencing at the clinical laboratory improvement amendments (CLIA)-approved Laboratory of Personalized Genomic Medicine at Columbia University Medical Center (New York, New York). Two patient samples (P2 and P4) were sequenced using a panel of 80 candidate genes by the CLIA-approved Blueprint Genetics laboratory (Helsinki, Finland). One patient sample (P5) was sequenced at the Casey Eye Institute (Portland, Oregon) using a panel of 211 genes. The predicted effects of variants were assessed for pathogenicity using in silico prediction software including PolyPhen-2, Mutation Taster, SIFT and Provean.

### Protein modeling

The online PHYRE2 server was used to generate the homology-based structural model of the BTB-BACK domains of KLHL7 (residues from 19 to 276) using the BTB-BACK domains structure of KLHL11 (PDB ID: 3I3N) as a template [34]. The crystal structure of KLHL7 Kelch domain (PDB ID: 3II7) was used to model mutants at the Kelch domain. Cullin binding to the BTB-BACK domains of KLHL7 was modelled using the crystal structure of KLHL11-Cul3 complex (PDB ID: 4AP2) as a template. The figure was generated using Pymol (The PyMOL Molecular Graphics System, Version 2.0 Schrödinger, LLC).

## Data Availability

The datasets used and/or analysed during the current study are available from the corresponding author on reasonable request.

## Abbreviations

adRP: Autosomal dominant retinitis pigmentosa
BA: Burian-Allen
BOS3: Bohring-Opitz cold-induced sweating syndrome-3
CLIA: Clinical Laboratory Improvement Amendments
CME: Cystoid macular edema
COST: Cone outer segment tips line
DTL: Dawson, Trick, Litzkow
ELM: External limiting membrane
EZ: Ellipsoid zone
ffERG: Full-field electroretinogram
ICC: Intraclass correlation coefficient
IRD: Inherited retinal dystrophy
NIR-AF: Near-Infrared autofluorescence
ONL: Outer nuclear fiber layer
RP: Retinitis pigmentosa
SD-OCT: Spectral domain optical coherence tomography
SW-AF: Short wavelength autofluorescence
UPP: Ubiquitin-proteasome degradation pathway
